# Human Erythrocyte Acetylcholinesterase in Health and Disease

**DOI:** 10.3390/molecules22091499

**Published:** 2017-09-08

**Authors:** Carlota Saldanha

**Affiliations:** Instituto de Bioquímica, Instituto de Medicina Molecular, Faculdade de Medicina, Universidade de Lisboa, Av. Professor Egas Moniz, 1649-028 Lisboa, Portugal; carlotasaldanha@medicina.ulisboa.pt

**Keywords:** acetylcholinesterase, red blood cells, nitric oxide

## Abstract

The biochemical properties of erythrocyte or human red blood cell (RBC) membrane acetylcholinesterase (AChE) and its applications on laboratory class and on research are reviewed. Evidence of the biochemical and the pathophysiological properties like the association between the RBC AChE enzyme activity and the clinical and biophysical parameters implicated in several diseases are overviewed, and the achievement of RBC AChE as a biomarker and as a prognostic factor are presented. Beyond its function as an enzyme, a special focus is highlighted in this review for a new function of the RBC AChE, namely a component of the signal transduction pathway of nitric oxide.

## 1. Introduction

Erythrocytes or red blood cells (RBC) are more than sacks of oxyhemoglobin or deoxyhemoglobin during the semi-life of 120 days in blood circulation [1]. Erythrocytes comport different signaling pathways which includes the final stage of apoptosis, also called eryptosis [2,3]. Exovesicules enriched with acetylcholinesterase (AChE) originated from membranes of aged erythrocytes appear in plasma [4]. Kinetic changes of the AChE enzyme have been observed in old erythrocytes [5]. Previously, AChE in erythrocytes was evidenced as a biomarker of membrane integrity [6]. Later on, increased impairment values of AChE enzyme activities were observed in several diseases as will be described below.

The blood physiological functions at macro- and microcirculatory vessel networks are dependent on RBCs’ membrane integrity and the normal interaction with endothelium and other blood components [7]. Luminal vascular endothelial cells can rest in a stationary phase or be activated during an inflammatory response. The degree of resolution of that response creates a solved acute inflammation or unsolved chronic inflammation. In all situations, erythrocytes are a player [8].

Depending on the degree of endothelium integrity, the plasma acetylcholine (ACh) induces vasodilation or vasoconstriction through the amount of nitric oxide (NO) synthesized by endothelial cells and released to the vessels smooth muscle [9,10]. The NO released from endothelial cells to the lumen is scavenged by the erythrocytes through the band 3 protein, providing a route for an NO influx to, and an efflux from, erythrocytes [11,12,13]. NO is rescued by the hemoglobin molecule forming *S*-nitrosohemoglobin (SNOHb) inside the erythrocyte [12,13]. In blood circulation, where the erythrocyte senses tissues with low partial oxygen pressure, NO is transferred from β3 SNOHb to the thiol group of band 3 with an NO efflux to the lumen vessel [13]. Using in vitro inhibitors of protein tyrosine kinase (PTK) and protein tyrosine phosphatase (PTP), phosphorylation and dephosphorylating of band 3 at tyrosine residues have been evidenced and the two forms exist in a dynamic equilibrium [14]. Dephosphorylate erythrocyte membrane band 3 is associated with oxyhemoglobin and with the glycolytic enzymes, glyceraldehyde dehydrogenase, aldolase, and phosphofructokinase, which disclose to the cytosol under phosphorylation band 3 state [15].

A higher erythrocyte aggregation tendency and increased membrane AChE enzyme activity is also evidenced when band 3 is phosphorylated, but not when it is dephosphorylated [16,17]. In addition, glutathione is an abundant molecule inside erythrocytes, which has a thiol group that can react with NO, forming nitrosothiols such as S-nitrosoglutathione (GSNO) [18]. The NO reservoir property attributed to glutathione might be influenced by the inactivation of glutathione reductase induced by the oxidative stress installed in erythrocytes [19].

Attempting to pursue the challenge of finding a physiological function for erythrocyte membrane AChE, the nitric oxide discovery triggered by plasma ACh gave us a clue about the action of AChE. Erythrocyte membrane AChE is involved in the nitric oxide (NO) signal pathway as evidenced, for the first time at the start of this century, in several in vitro studies using blood samples from healthy donors as described below.

## 2. Biochemical Properties of Human Erythrocyte Membrane Acetylcholinesterase

Human erythrocyte acetylcholinesterase (AChE) discovered by Alles and Haves in 1940 was later, in 1961, classified as EC.3.1-1.7 by the Enzyme Commission [20,21]. Only in 1975 the appropriate process of extraction and purification of the erythrocyte membrane AChE confirmed it as a glycoprotein [22]. Later in 1985 it was shown that this enzyme, located in the external leaflet of the erythrocyte membrane, is a dimeric protein [23]. The catalytic efficiency of the dimeric form of AChE depends on the amphipathic medium of extraction and purification [24,25]. AChE belongs to the glycosylphosphatidylinositol (GPI)-anchored protein family and bears the Yta blood group antigen [26,27].

**Figure 1:**
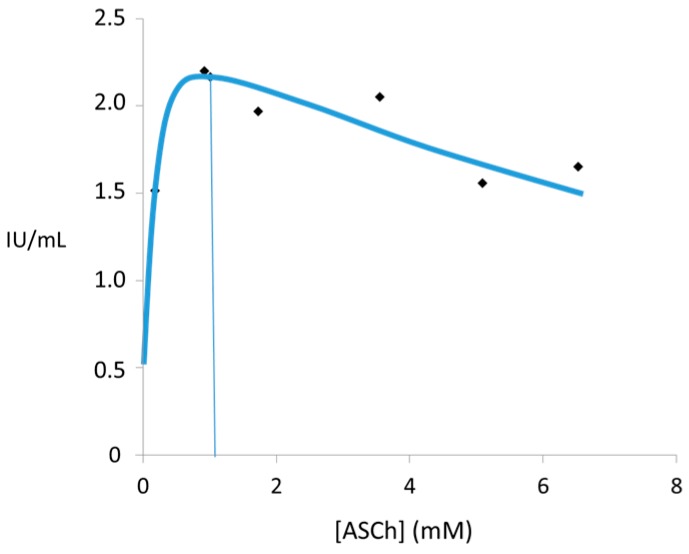
Bell-shape curve of enzymatic hydrolysis of hydrolysis of acetylthiocholine (ASCh) by AChE purified from human erythrocytes indicate the inhibition of the reaction by the substrate. Applying the Ellman’s method, AChE 0.04 IU/mL was added to preincubated for 10 min, at 25 °C, in 1 mL assay solutions with 100 mM PO_4_ buffer, pH 8.0, and 0.2 mM DTNB [5,5-dithiobis(2-nitrobenzoic acid)] before adding the substrate at 0.0025 to 6 mM final concentrations. All experiments were repeated three times, and similar results were obtained. All the values obtained above 1 mM of ASCh were diminished with those obtained in non-enzymatic hydrolysis. All data were analyzed applying the least square method to the velocity equation coupled with the iterative non-linear method [24].

The kinetic profile of AChE shows a bell-shape curve, (Figure 1) [24], meaning that the enzyme-free, enzyme substrate complex and the acyl enzyme intermediate form all exist in the reaction medium; AChE is uncompetitive, inhibited by the excess of substrate concentration which has been shown also by others authors [28]. This mechanism showed a second substrate molecule binding to the free anionic group of the active center of the acyl enzyme complex [24]. The optimum substrate concentrations values, which result from the velocity curve profile, are dependent on the native, solubilized, or purified forms of the enzyme, of the ionic strength of the medium and pH values and of the type and concentration of inhibitor or activator compounds [24]. Another parameter influent on optimum substrate values is the method applied for the kinetic evaluation according to the product, thiocholine or acetate, that needs to be measured when acetylthiocholine is used as the synthetic analog of acetylcholine (ACh), the natural substrate of AChE [24,29]. A lower affinity constant for the substrate and a higher optimal substrate concentration value are obtained at high ionic strength in relation to the lower ionic strength of the buffer medium [24].

The decrease of acylation velocity constant values with increased substrate concentrations were obtained by a stopped-flow technique at low ionic strength using the purified form of human erythrocyte AChE, thus confirming an uncompetitive inhibition by the substrate [30]. Due to the high AChE enzyme activity at high ionic strength, the stopped-flow technique cannot be applied in the transient phase of the acylation reaction [30].

Later, the same bell-shape kinetic profile curve was observed in krait venom AChE, the inhibition by substrate concentration being reduced using a high ionic strength buffer in relation to low ionic strength, as has been described for human erythrocyte AChE [24,31].

Large amounts of AChE are present in erythrocyte membrane exovesicles [32,33]. A biochemistry laboratory practice for students, based on phthalate gradient concentrations was described using erythrocyte suspensions to obtain aggregates of exovesicles, which are characterized by AChE enzyme activity, protein, and phospholipids coloration processes [33]. This laboratory experimentation matches objectives with teaching and learning activities in biochemistry courses [33]. The same experimental protocol when used in erythrocyte suspensions under the presence of the membrane fluidity fluorescent probes (diphenylhexatriene) DPH or trimethylamino-dipheny-lhexatriene (TMA-DPH) or heptadecyl-hydroxycoumarin (C17-HC) showed the presence of exovesicles in all the supernatants, confirmed by the AChE enzyme activity that is higher in those incubated with the hydrophobic probe DPH in relation to the others amphiphilic TMA-DPH and C17-HC [34]. At variance, the intensity and fluorescence anisotropy is higher in TMA-DPH and C17-HC supernatants vesicles in relation to those obtained from the DPH [34]. Therefore, amphiphilic probes TMA-DPH and C17-HC are preferentially incorporated in the exovesicles when compared with DPH [34]. The exovesiculation process can, for example, occurs during the transformation of discocyte to stomatoscyte shape in the presence of certain types of therapeutic drugs and oxidative stress [35,36]. Mathematical modeling applied to those switch RBC forms can contribute to understanding, in patients under drug therapy, the biorheogical behavior of RBCs in tissue oxygenation [37]. RBCs’ AChE enzyme activity and osmotic fragility assessment are valuable for testing the cytotoxicity degree of lipophilic drugs that may disrupt cellular membranes beyond their final usefulness, for example, as a cell nucleus target [6].

## 3. Implications of Erythrocyte Membrane Acetylcholinesterase Enzyme Activity in Disease

In 1973 erythrocyte AChE enzyme activity was reported as a marker of membrane integrity [38].

Changes in RBC AChE enzyme activity were evidenced in health and disease states. Reduced erythrocyte AChE activity in aged humans and in neonates related to adult man was evidenced [5,38]. Sub-fractions of RBCs of different ages, prepared in vitro, showed that the oldest RBCs present an AChE enzyme activity that is lower than the young RBC subpopulations, erythrocyte AChE being considered a biomarker of aging [39]. Increased RBC AChE enzyme activity has been evidenced in blood samples taken from healthy females [40].

In healthy females, adrenaline decreases AChE activity when α- and β-adrenergic receptors are blocked and an inverse significant correlation between erythrocyte membrane rigidity and AChE activity has also been registered. In erythrocytes from healthy males, adrenaline increases AChE activity when no adrenergic receptors are blocked [5]. An opposite profile of erythrocyte membrane fluidity under an adrenaline effect was observed in relation to that obtained in AChE activity in both genders [5]. Peripheral blood from males and females has shown echinocytes when adrenaline is present [5].

The discovery of sex-related differences in erythrocyte AChE activity and in the membrane hydrophobic region fluidity under the adrenaline influence can contribute to understanding different responses, attitudes, and behaviors with respect to stress situations, usually verified in both genders. The existence of certain gender characteristics, at the cellular level, has important implications in disease and medication responses.

Lower RBC AChE enzyme activity among farmers exposed to pesticides has been reported [41]. A recent evaluation of longitudinal changes of AChE and paraoxonase-1 enzyme activities in greenhouse workers, over a crop season, reveals that the decrease in RBC AChE results from an indirect effect of pesticides, generating oxidant molecules, inducing lipid peroxidation, and consequently interfering with the erythrocyte membrane’s integrity [41,42].

Patients with paroxysmal nocturnal hemoglobinuria and others with hemolytic anemia have been characterized by lower levels of erythrocyte AChE [5].

In non-insulin diabetes mellitus patients undergoing routine angiography, an impairment in RBC AChE enzyme activity and a lower fluidity in the hydrophobic erythrocyte membrane domain after fluorescein injection was verified [43]. The less active state of AChE probably results from conformational molecular changes occurring in AChE due to its tail insertion nearby or on high rigidity membrane domains [43].

Ex vivo studies using blood samples obtained from patients suffering different diseases, namely Parkinson, essential hypertension, glaucoma, retinal vasculitis, amyotrophic lateral sclerosis (ALS), and Hirschsprung’s disease, have evidenced augmented levels of RBC AChE enzyme activity [44,45,46,47,48,49]. Erythrocyte AChE is considered a biomarker of essential hypertension, glaucoma, ALS, neurotoxicity, and pesticide poisoning and a diagnostic marker in Hirschsprung’s disease [45,46,48,49,50,51,52]. Higher AChE enzyme activity in RBCs were verified in glaucoma, essential hypertension, and ALS, which are inflammatory vascular diseases characterized by a presence in the blood of high inflammatory molecule concentrations, reactive oxygen species, and reactive nitrogen species [45,46,49,50,53,54]. Consequently, erythrocyte AChE is considered a marker of inflammation [54,55,56]. The modulation of AChE enzyme activity by its natural substrate acetylcholine or by a strong inhibitor, such as velnacrine, showed ACh with an anti-inflammatory effect characterized by its protective action before inflammation development [56]. These anti-inflammatory characteristics of ACh were observed in vivo, by intravital microscopy, in an experimental animal model, by the quantification of pro-inflammatory cytokines production and by the visualization and quantification of leukocyte recruitment which includes the number of rolling and adherent leukocytes and their rolling velocities [55].

## 4. Enrolment of Erythrocyte AChE in the Signal Transduction Pathway of Nitric oxide

Vascular endothelial cells change its phenotypes to participate in the acute inflammation, which involves a faster or slower response in close relation to white blood cells [57]. Fibrinogen and the ACh levels among other inflammatory molecules increase in plasma [58,59].

**Figure 2:**
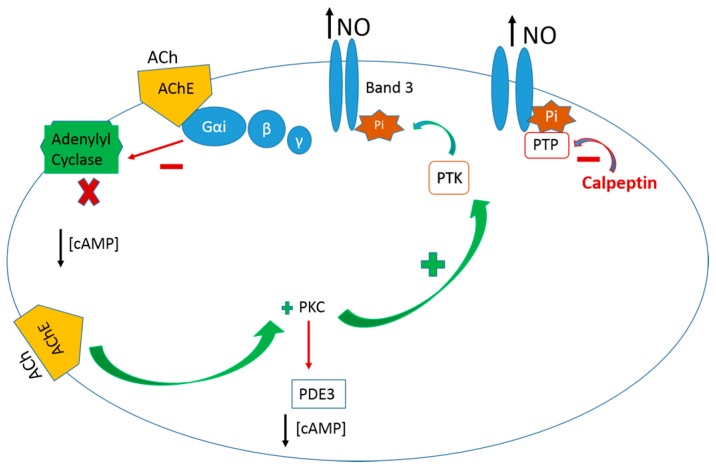
Schematic representation of the AChE–ACh active complex transduction pathway of the NO efflux from human erythrocytes supported by in vitro studies, as explained in the text [17,61,64,65,66].

It has been shown in vitro that erythrocytes, in the presence of ACh, the natural substrate of the membrane AChE, are able to release NO, which can be quantified by an amperometric method with an amiNO-sensor [8,60]. It was also verified that the AChE–ACh enzyme active complex activates the protein kinase C (PKC), which phosphorylates the protein tyrosine kinase (PTK) transforming it from an inactive to an active state by covalent modulation (Figure 1) [61,62]. The amount of NO is reinforced by calpeptin, a PTP inhibitor (Figure 1) [61]. PTK phosphorylates membrane band 3 protein, which becomes able to receive NO in its thiol group from *S*-nitrosohemoglobin molecules, allowing the efflux of NO from erythrocytes [61]. Erythrocyte PKC phosphorylates protein tyrosine phosphatase (PTP) and PI3K phosphorylates phosphodiesterase-3 (PDE3) (Figure 1) [62,63]. Consequently, neither band 3 protein is dephosphorylated nor is PDE3 blocked to hydrolyze cyclic adenosine triphosphate (cAMP) (Figure 2) [63,64]. It was evidenced that the AChE–ACh enzyme active complex, joined with the Gα_i_ protein, inhibits adenylyl cyclase (AC) making it unable to generate cAMP from adenosine triphosphate (Figure 1) [17]. Another in vitro study showed that, by inducing active protein conformations in erythrocyte AChE, by band 3 phosphorylation, the NO efflux from erythrocytes increases (Figure 2) [65].

The NO efflux measurements from erythrocytes are based on the ACh signal transduction pathway through the AChE–ACh active enzyme complex in active conformation, associated with Gα_i_ protein/AC, the phosphorylation of band 3 protein (PTK), PKC and PI3K/PDE3 proteins, and cAMP molecules (Figure 2) [17,61,64,65,66].

Studies in vivo with erythrocyte suspensions were also done either in the presence of velnacrine or timolol, which are, respectively, strong and moderate AChE inhibitors [17,61,63,67,68]. In the presence of velnacrine, the efflux of NO from erythrocytes is lower than that in the presence of ACh; meanwhile, at variance, GSNO and peroxinitrite concentrations quantified in erythrocytes under the AChE–velnacrine inactive enzyme complex were higher than those obtained in the presence of the erythrocyte AChE–ACh complex [63,66]. The erythrocyte band 3 protein phosphorylation was absent in erythrocytes under the presence of velnacrine [63]. It was also evidenced by an in vivo study that AChE active or less active molecular conformations induces an increase or decrease in NO efflux from erythrocytes, respectively [69].

**Figure 3:**
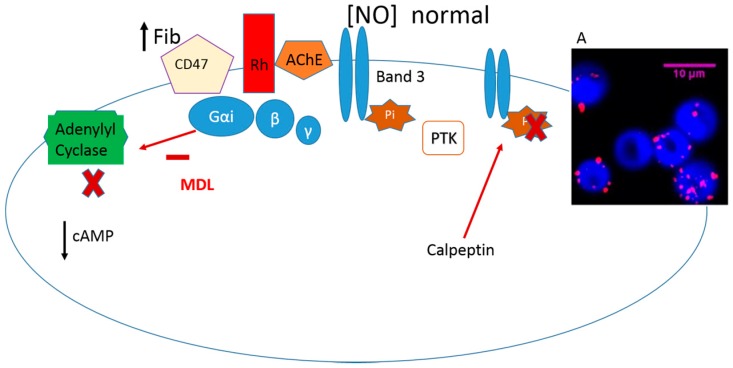
Schematic representation of the soluble fibrinogen transduction pathway of NO efflux from human erythrocytes supported by in vitro studies of hyperfibrinogenemia model under the presence of calpeptin inhibitor of PTP [70,71,72,73]. A: The soluble fibrinogen binding to erythrocyte membrane (blue color) CD47 (red color) [73].

When hyperfibrinogenemia is mimicked in vitro, the amount of NO efflux from erythrocytes increases, depending on band 3 protein phosphorylation and low cAMP levels (Figure 3) [70,71,72,73]. It was evidenced that, in the same model of hyperfibrinogenemia, the NO efflux returns to normal levels in the presence of ACh, showing its dependence on AChE conformational states. This influence results from the fibrinogen binding to CD47 of RH complex, associated with Gα_i_ protein, which influences the membrane enzyme AChE molecular conformational states (Figure 3A) [73].

Previous studies under soluble fibrinogen at physiological concentration evidence a lower NO efflux from erythrocytes, which indicates the scavenger RBCs’ ability, confirmed by the increased of GSNO levels [74].

When the inhibitor of the erythrocyte Casein Kinase 2 (a cytosol protein that phosphorylates band 3 protein) is present in the erythrocytes suspensions at high fibrinogen concentration, the NO efflux is maintained as normal, confirming the dependence on band 3 phosphorylation [75,76].

Interestingly, the forskolin, an activator of the AC enzyme, normalizes the levels of NO efflux from erythrocytes in an in vitro model of hyperfibrinogenemia, and it is nowadays used to alleviate symptoms of glaucoma [77,78]. Glaucoma patients present an increase in nitrogen reactive species in aqueous humor, and the NO efflux from their erythrocytes is higher than that in healthy humans [12,79]. Therefore, one possible explanation for the therapeutic success of forskolin is the ability of erythrocytes to preserve NO, preventing it from combining with oxygen and generating reactive species formation. This is a clue to be explored in patients taking forskolin medication, where high levels of fibrinogen have been observed.

## 5. Conclusions

The erythrocyte membrane AChE’s enzyme activity values are implicated as a biomarker of membrane integrity (normal), aging (lower), gender (higher in females than in males), inflammation (higher), neurotoxicity (higher), and pesticide poisoning (higher). The erythrocyte AChE is used as diagnostic marker in Hirschsprung’s disease. The active state of AChE is modulated by the membrane band 3 protein phosphorylation, meaning that this enzyme activity can be manipulated from inside the erythrocytes.

Erythrocyte AChE is a biomarker of inflammation and is involved in the white blood cells approaches to the endothelial vessel wall and in the production of pro-inflammatory cytokines.

At microcirculation, blood flow through small vessels favors gas exchanges, such as the exchange between oxygen and nitric oxide with carbon dioxide, delivers nutrients metabolites, and removes waste products. Erythrocytes deliver NO in tissues with lower oxygen partial pressure (PaO_2_) and scavenge it at high PaO_2_ through the band 3 protein [80]. The ability of erythrocytes to deliver or retain NO depends of the membrane integrity, of the AChE activation state and of its molecular protein conformations.

The signal transduction pathways associated with NO mobilization in erythrocytes were described under the influence of the endogenous plasma compounds, namely, ACh and fibrinogen, whose levels increase in inflammation. There are specific key points in those pathways where activators or inhibitor molecules of AChE, PTK, PTP, AC, PDE3 and PKC change the NO efflux from erythrocytes, which, in the future, might be considered as therapeutic targets in vascular inflammatory diseases.

It is mandatory to highlight the new function for erythrocyte enzyme membrane AChE acting as receptor for hydrophilic blood circulating molecules in the NO signal transduction pathway.
